# Characterization of occupational, demographic and health determinants in Canadian reservists veterans and the relationship with poor self-rated health

**DOI:** 10.1186/s12955-020-01516-8

**Published:** 2020-08-10

**Authors:** Julian Reyes, Jill Sweet, MaryBeth MacLean, Alain Poirier, Linda VanTil

**Affiliations:** Veterans Affairs Canada, Charlottetown, PE C1A 1N2 Canada

**Keywords:** Reserve force, Veterans, Canada, Self-rated health, Survey, Risk factors

## Abstract

**Background:**

Self-rated health is an useful indicator of the general health in specific populations and used to propose interventions after service in the military context. However, there is scarce literature about self- rated health (SRH) in the Canadian Veterans of the Reserve Force and its relationship with demographic, health and occupational characteristics of this specific group. The aims of this research were to determine the SRH in Canadian Reserve Force Veterans and to explore the relationship between demographic, military service and health factors by reserve class.

**Methods:**

Data from the individuals was collected from the Life After Service (LASS) 2013 survey, including Veterans with Reserve Class C (*n* = 922) and Class A/B (*n* = 476). Bivariate and multivariate analysis using logistic regression models, were used to assess the association between the demographic characteristics, physical health, mental health, and military service characteristics and the self-rate health by both reserve classes.

**Results:**

The overall prevalence of poor SRH in Reserve Class C Veterans was 13.1% (CI:11.08–15.4) and for Reserve Class A/B was 6.9% (CI:5.0–9.1). Different degrees of associations were observed during the bivariate analysis and two different models were produced for each reserve class. Veterans of Reserve Class C showed that being single was (OR = 2.76, CI: 1.47–5.16), being 50–59 years old (OR = 4.6, CI: 1.28–17.11), reporting arthritis (OR = 2.49, CI: 1.33–4.67), back problems (OR = 3.02, CI:1.76–5.16), being obese (OR = 1.96, CI: 1.13–3.38), depression (OR = 2.34, CI: 1.28–4.20), anxiety (OR = 4.11, CI: 2.00–8.42), PTSD (OR = 2.1 CI: 0.98–4.47), PTSD (OR = 20.9, CI:0.98–4.47) and being medically released (OR = 4.48, CI: 2.43–8.24) were all associated with higher odds of poor SRH. The Reserve Class A/B model showed that completing high school (OR = 4.30, CI: 1.37–13.81), reporting arthritis (6.60, CI: 2.15–20.23), diabetes (OR = 11.19, CI: 2.72–46.0), being obese (OR = 3.37, CI: 1.37–8.27), daily smoking (OR = 2.98, CI: 1.05–8.38), having anxiety (OR = 9.8, CI: 3.70–25.75) were associated with higher odds of poor SRH.

**Conclusions:**

These results suggested that the relationship of poor SRH with demographic, health and military occupation domains varied depending on the class on the Reserve Force Service. Different strengths of association showed different risk compositions for both populations. This can be used to better understand the health and well-being of Veterans of the Reserve Force.

## Background

The assessment of the general health status and quality of life has been considered a one of the elements of the well-being of the individuals in a population. Self-rated health (SRH) is a commonly used indicator in the psychological and clinical fields in population surveys and is a measure where the individuals rate their own health, as a single question with responses such as: “excellent”, “very good”, “good”, “fair” or “poor” [1]. The self-rated health (SRH) contains a continuum of the health/illness concepts, ranging from positive (absence of illness) to negative concept (not bad to neutral state) [2]. The SRH has been considered as the sum of the person’s health status and functional outcomes [2] and a good descriptor of the general health and well-being for a particular population and a predictor of morbidity, mortality [3] and health services utilization [4]. This indicator has been associated with psychological health at an individual level [5], and to chronic health conditions and to acute or transitory illnesses on a lesser degree [6, 7]. Moreover, the SRH measured in adults has shown high correlation with complex health indices, showing the validity of this construct [8].

Different studies have assessed the determinants of SRH in a wide variety of population backgrounds, ranging from minorities, populations with disabilities and developing countries [2, 9] and amongst active and retired military members [10–13]. Furthermore, military occupation adds a level of complexity in physical and mental health measurements, since personnel with military service are occupationally exposed to hazardous activities, such as heavy machinery, hazardous activities and unpredictable environments [14]. In personnel transitioning from military service to civilian life, the assessment of different dimensions of well-being have been explored, including the SRH assessment [12].

Multiple demographic factors, social support, marital status, income and occupation amongst others, identified as contextual factors, have been reported in military populations by different authors as having an effect over the functional health status [10, 15] . One study showed, that self-reports of low health status were associated with higher health utilization following a major deployment [10]. In Canada, the Life After Service Studies (LASS) program designed to increase the understanding of the transition from military to civilian life, has described the well-being of Veterans, including health status and other factors, in Canadian Veterans released since 1998 [16]. This study used the second cycle of LASS from 2013, since this cycle expanded data collection to include Veterans of both Regular Force and the Primary Reserve Force, providing the opportunity to explore more health aspects of this component of the Canadian Forces [17]. Furthermore, a characterization of the Canadian Reserve Force, has been reported describing outcomes such as: income, occupation, community-family support, health-disability and mental health [17]. This study showed, that between 65 to 90% of the Reserve Veterans with different classes of service presented above average quality of life for physical health [17]. There are differences in the Canadian Forces components (Regular and Reserve Force) due to the nature of service (for example, deployment, combat or domestic responsibilities) and enrollment options (full time vs part time), that could influence the perception of health and the distribution of specific factors. These differences could be carried over the transition to the civilian life. For both components the adaptation to civilian life after a military career can be a challenging event that could exacerbate service or non-service related physical and psychological difficulties [18]. However, there is scarce literature exploring the association of various factors with poor SRH, specifically amongst Reserve Force Veterans and further understanding is needed in this Canadian Forces component after transitioning to civilian life. Therefore, the objectives of this study were to assess the SRH of Veterans of the Canadian Reserve Force, and to identify the association with the demographic, military service and health factors.

## Methods

This research used the LASS 2013 computer assisted cross sectional survey developed by the Research Directorate of Veterans Affairs Canada conducted by Statistics Canada in February–March 2013 [16, 17]. This initiative consisted in a population health research program with the aim of improving the health of Veterans in Canada, through the understanding of the ongoing effects of military service [16]. Canadian Veterans were defined as former members of the Canadian Armed Forces from both components (Regular or Reserve) regardless of their length of service, and their status as clients of VAC [16]. This study assessed only Veterans of the Reserve Force.

### Data source

The data collection for this analysis was conducted as part of the Life After Service (LASS) 2013 study, which included those with service in the Canadian Forces since 1998 [16, 17]. The survey consisted in a computer assisted telephone survey conducted by Statistics Canada including 193 questions covering different modules about health disability and health determinants. The population frame included those with service in the Canadian military since 1998. The survey reached 4149 Canadian Veterans with a 70% response rate and with 90% of agreement to share the information [19]. The methods of this survey have been described previously in technical reports of the Research Directorate [19].

### Reserve force classification

The Reserve Force was composed of professional members of the Canadian Armed Forces with voluntary service, not required for deployment and with part-time and non-continuous employment enrollments [17]. The Reserve Force Veterans were classified as Class C or Class A/B. Class C were deployed domestically or internationally on a period of full-time service in the Reserve Force between 2003 and 2012, also had periods of Class A and Class B service and no Regular Force service. Class A/B had a combination of periods of both Class B (temporary full-time) service and Class A (part-time) service between 2003 and 2012, with no periods of Class C or Regular Force service.

### Questionnaire description

The questionnaire included: health status, chronic health conditions, disability, labor force status, social support and health care utilization, as well as some military-specific questions. Four domains such as demographic characteristics, physical health, mental health, and military service characteristics, were selected for the present analysis and were extracted from the LASS 2013, covering 14 indicators and were included in this analysis. A detailed description of the variables is presented in the Methodology: Life After Service Studies 2013 [19].

### Demographic variables

The demographic domain included categorical variables for age at release from service (< 30, 30–39, 40–49, 50–59, > 60), sex (male and female), education level (less than high school, high school and university degree) and marital status (married or common law and single).

### Chronic health characteristics

Physical health included chronic condition diagnosis (yes or no response) to any of the following conditions: asthma, respiratory (asthma or COPD), diabetes, arthritis, back problems, obesity, cancer, hypertension, hearing impairment. Also measured were life style behaviors such as daily smoking and heavy drinking (five or more drinks per occasion, at least once a month).

### Outcome variable

The SRH was selected as the outcome variable (excellent, very good, good, fair or poor responses). The responses were categorized into two: excellent/very good/good as the reference category (0) and fair/poor as the risk category (1). The choice of this dichotomization was due to the unbalanced frequency of the original responses. The interest in the present analysis is to high light the difference between those who are doing fair or poor against those categorized as doing well.

### Mental health

The mental health domain module from the LASS 2013 survey included the Self-Perceived Mental Health, corresponding to the question: in general would you say your mental health is?, and was composed of the answer categories: Excellent/Very Good/Good and Fair/Poor. Other variables considered in the analysis were depression (diagnosed with major depression, bipolar disorder, mania or dysthymia), post-traumatic stress disorder, and anxiety disorder (diagnosed with phobia, OCD, panic disorder).

### Military service characteristics

The military service characteristics assessed were length of service (< 2 years, 2 to 9 years and 10 to 19 years), enrollment year (1950/60s, 1970s, 1980s, 1990s, 2000s and 2010s), last rank (Officers, Senior Non-commissioned Members (NCM) and Junior NCM), environment (air, land and sea), release type (Involuntary, Medical, Voluntary-Retirement/Service Complete) and military occupation at release (MOC) (combat arms, communications, maritime, aviation, administrative /logistics, engineering/technical, medical and Generalized Operational Services (GOS).

### Statistical analysis

A descriptive analysis of the categorical and continuous variables was performed adjusting for the complex stratified design of the survey. Moreover, a bivariate analysis, for each one of the predictors were performed to explore the frequency distribution of the variables and the relationship with SRH status. A statistical significance of *P* < 0.1 was used to select the predictors in the bivariate analysis using a weighted logistic approach to be further considered in the multivariate analysis.

The two Reserve classes were considered separately for the analysis due to their very different service characteristics. Veterans of Reserve Class A/B had mostly part time service, whereas Reserve Class C Veterans had full time service at one point of their career and had other similarities with the Regular Force. In addition, one of the design strata for LASS 2013 survey was the Reserve Class (Reserve A/B and C). A weighted logistic regression model was calculated, assessing first for the significance within each domain of variables using a backward elimination process and then testing the significant variables together for both classes, with a statistical significance of *P* < 0.05 used to select the variables for the final model.

Wald tests of composite linear hypotheses for independent variables with more than two categories were performed to explore the overall statistical significance for all categories. Moreover, the potential correlation between predictors, interaction and confounding effects were assessed, correlation of the regression coefficients, testing interaction terms and stratified analysis were explored. All the statistical analyses were carried out in STATA version 13.

## Results

The total number of Reserve Class C Veterans was 922 and 476 Class A/B Veterans who participated in the LASS 2013 survey and agreed to share their information with Veterans Affairs Canada. The distribution of the variables of interest by each domain can be found in Table 1. Mainly, Reserve Class C Veterans were older and married compared to Reserve Class A/B Veterans. Also Reserve Class C Veterans reported more arthritis and back problems, served for 10 years or more, had administrative occupations, and were discharged for medical reasons compared with the Reserve Class A/B Veterans (Table 1).

**Table 1:** Univariate weighted analysis of Demographic, physical health, mental health and military service characteristics of Reserve Force Veterans, by class

Domain	Variable	Category	Reserve Class C			Reserve Class A/B		
			n	%	CI	n	%	CI
Demographics	Gender of respondent	Male	703	76.6	(73.8–79.3)	387	81.5	(77.7–84.7)
		Female	219	23.4	(20.8–26.3)	89	18.5	(15.3–22.3)
	Age	< 30	135	17.1	(14.6–19.9)	253	56.5	(52.0–60.9)
		30–39	366	40.6	(37.4–43.9)	139	27.7	(23.9–31.9)
		40–49	184	18.7	(16.4–21.4)	50	9.9	(7.6–12.9)
		50–59	150	15.2	(13.1–17.7)	17	3.0	(1.9–4.9)
		60+	87	8.4	(6.8–10.2)	17	2.8	(1.8–4.5)
	Education	Less than HS	28	3.1	(2.1–4.5)	10	1.9	(1.0–3.5)
		High School Grad	234	25.9	(23.2–28.9)	115	25.0	(21.3–29.2)
		Post Sec < Bach	376	40.9	(37.7–44.1)	182	38.5	(34.1–43.0)
		Post Sec > = Bach	283	30.1	(27.2–33.2)	168	34.6	(30.5–39.1)
	Marital Status	Married/Common-Law	673	71.8	(68.7–74.7)	272	56.3	(51.8–60.8)
		Sep/Wid/Div	60	6.2	(4.8–7.9)	21	4.3	(2.8–6.5)
		Single,Never Married	189	22.0	(19.4–25.0)	182	39.4	(35.0–43.9)
Physical Health	Arthritis	Yes	158	16.1	(13.9–18.6)	31	5.9	(4.1–8.3)
		No	757	83.9	(81.4–86.1)	444	94.1	(91.7–95.9)
	Back Problems	Yes	297	31.5	(28.6–34.6)	84	17.0	(13.9–20.6)
		No	623	68.5	(65.4–71.4)	392	83.0	(79.4–86.1)
	Respiratory	Yes	68	7.3	(5.8–9.2)	26	5.6	(3.8–8.1)
		No	851	92.7	(90.9–94.3)	449	94.4	(91.9–96.2)
	Diabetes	Yes	50	4.9	(3.7–6.4)	8	1.3	(0.6–2.6)
		No	870	95.1	(93.6–96.3)	468	98.7	(97.4–99.4)
	Obese	Yes	219	23.5	(20.8–26.4)	86	17.8	(14.6–21.6)
		No	689	76.5	(73.6–79.2)	385	82.2	(78.4–85.4)
	Cancer	Yes	10	1.0	(0.5–1.9)	4	0.8	(0.3–2.2)
		No	910	99.0	(98.1–99.5)	471	99.2	(97.7–99.7)
	High Blood Pressure	Yes	121	12.3	(10.4–14.6)	33	6.1	(4.4–8.6)
		No	796	87.7	(85.5–89.6)	440	93.9	(91.4–95.6)
	Hearing	Yes	45	4.7	(3.5–6.3)	7	1.3	(0.6–2.8)
		No	847	95.3	(93.7–96.5)	464	98.7	(97.2–99.4)
	Smoker	Daily Smoker	120	13.4	(11.3–15.9)	49	10.3	(7.8–13.4)
		Not Daily Smoker	802	86.6	(84.1–88.7)	427	89.7	(86.6–92.2)
	Heavy Drinking	Yes	244	27.8	(24.9–30.9)	149	31.7	(27.6–36.1)
		No	671	72.2	(69.1–75.1)	323	68.3	(63.9–72.4)
Mental Health	Self-Rated Mental Health	Excellent/VG	618	67.2	(64.1–70.2)	348	73.7	(69.5–77.5)
		Good	200	22.0	(19.4–24.9)	97	19.9	(16.6–23.8)
		Fair/Poor	103	10.8	(8.9–12.9)	31	6.4	(4.5–9.0)
	Depression	Yes	114	12.1	(10.2–14.4)	29	5.8	(4.0–8.3)
		No	807	87.9	(85.6–89.9)	446	94.2	(91.7–96.0)
	Anxiety	Yes	76	8.1	(6.5–10.0)	26	5.5	(3.7–7.9)
		No	843	91.9	(90.0–93.5)	449	94.5	(92.1–96.3)
	PTSD	Yes	71	7.5	(5.9–9.3)	9	1.8	(0.9–3.4)
		No	842	92.5	(90.7–94.1)	464	98.2	(96.6–99.1)
Military Characteristics	Length of Service	< 2 years	7	0.9	(0.4–1.9)	95	20.6	(17.1–24.5)
		2 to 9 years	338	41.1	(37.9–44.4)	306	66.0	(61.6–70.1)
		10 to 19 years	352	36.4	(33.3–39.5)	55	10.1	(7.8–13.0)
		> = 20 years	225	21.6	(19.2–24.3)	20	3.3	(2.2–5.2)
	Enrollment	1950–80s	249	24.2	(21.6–27.0)	25	4.4	(3.0–6.5)
		1990s	444	48.0	(44.8–51.3)	86	16.1	(13.2–19.6)
		2000–10s	229	27.8	(24.8–30.9)	365	79.4	(75.7–82.7)
	Rank	Officers	169	17.2	(14.9–19.7)	51	10.2	(7.8–13.2)
		Senior NCM	204	20.4	(18.0–23.1)	22	4.0	(2.6–6.0)
		Junior NCM	549	62.4	(59.2–65.5)	403	85.9	(82.5–88.7)
	Environment	Air	67	6.9	(5.4–8.7)	19	3.5	(2.2–5.5)
		Land	738	80.4	(77.7–82.9)	394	83.3	(79.7–86.4)
		Sea	117	12.7	(10.7–15.1)	63	13.2	(10.4–16.6)
	Release Type	Involuntary	69	9.9	(7.9–12.3)	62	15.7	(12.4–19.5)
		Medical	128	12.5	(10.6–14.7)	17	3.2	(2.0–5.0)
		Vol/Ret/Service Complete	725	77.6	(74.7–80.3)	395	81.1	(77.2–84.7)
	Military Occupation	Combat arms	282	43.8	(40.0–47.7)	226	59.1	(54.2–63.9)
		Communications	71	10.8	(8.7–13.5)	26	6.4	(4.4–9.3)
		Maritime	51	8.0	(6.1–10.4)	39	9.9	(7.3–13.3)
		Aviation	11	1.5	(0.9–2.8)	4	0.8	(0.3–2.2)
		Admin	187	27.0	(23.8–30.6)	60	14.9	(11.7–18.7)
		Engineering	27	3.7	(2.5–5.3)	9	2.3	(1.2–4.4)
		Medical	27	4.0	(2.7–5.8)	23	5.5	(3.6–8.1)
		GOS	8	1.1	(0.5–2.2)	5	1.1	(0.5–2.6)
Outcome Variable	SRH	Excellent/VG	554	61.2	(58.0–64.3)	327	69.3	(65.0–73.4)
		Good	241	25.7	(23.0–28.7)	114	23.7	(20.0–27.7)
		Fair/Poor	127	13.1	(11.1–15.4)	35	7.0	(5.0–9.6)

**Fig. 1:**
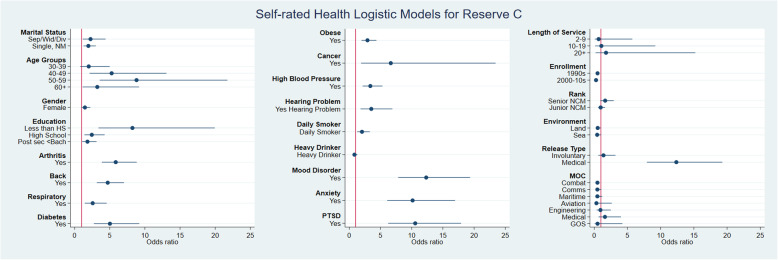
Bivariate analysis for SRH Demographic, physical health, mental health and military service variables in Reserve Forces Class C

**Fig. 2:**
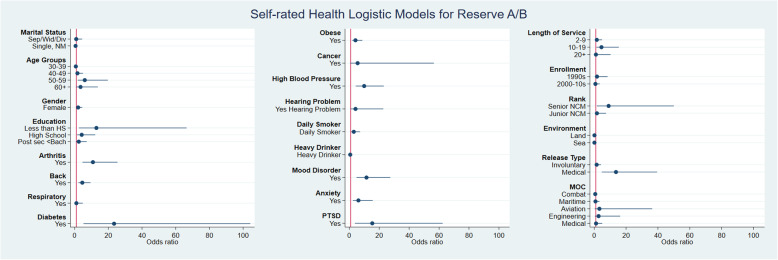
Bivariate analysis for SRH Demographic, physical health, mental health and military service variables in Canadian Reserve Forces Class A/B

The bivariate analysis is shown in Fig. 1 and Fig. 2 (further detail in the supplementary Tables 1 and 2) for both Reserve Force classes. Different degrees of association were observed for the independent variables. Reserve Class C Veterans had the following independent variables that met the selection criteria for the SRH model: demographic (gender, age, education and marital status), physical health (arthritis, back problems, respiratory, diabetes, obesity, cancer, high blood pressure, hearing and smoking) mental health (depression, anxiety and PTSD), military service (enrollment era, rank, environment, release type and MOC). Reserve Class A/B Veterans had a slightly different list of statistically significant variables: demographics (gender, age, education), physical health (arthritis, back problems, diabetes, obesity, high blood pressure, hearing and smoking), mental health (depression, anxiety, PTSD) military service (length of service, enrollment, rank, environment and release type).

Two different multivariate logistic models were assessed for each Reserve class. These results are presented in Tables 2 and 3. Eleven independent variables were included in both models with different magnitudes of association between the exposure and SRH. Reserve Class C Veterans included marital status, age, arthritis, back problems, obesity, depression, anxiety, PTSD, type of release. On the other hand, Reserve Class A/B Veterans included education, arthritis, diabetes, obesity, daily smoking and anxiety. In both models the variables arthritis, obesity and anxiety were common.

**Table 2:** Multivariate Logistic model for Self-Rated Health of Veterans of Reserve Class C (*n* = 891)

Variables	Odds Ratio	Std. Err.	P	95% Conf Interval	
				IL	UL
Marital Status					
Sep/Wid/Div	1.04	0.55	0.929	0.37	2.94
Single, NM	2.76	0.88	0.001	1.47	5.16
Age					
30–39	1.90	1.09	0.261	0.61	5.86
40–49	2.70	1.75	0.124	0.76	9.60
50–59	4.60	3.00	0.019	1.28	17.11
60+	2.90	2.10	0.144	0.69	12.52
Arthritis					
Yes	2.49	0.79	0.004	1.33	4.67
Back Problems					
Yes	3.02	0.82	0.000	1.76	5.16
Obesity					
Yes	1.96	0.54	0.016	1.13	3.38
Depression					
Yes	2.34	0.71	0.005	1.28	4.20
Anxiety					
Yes	4.11	1.50	0.000	2.00	8.42
PTSD					
Yes	2.09	0.81	0.055	0.98	4.47
Release Type					
Involuntary	1.23	0.60	0.672	0.46	3.20
Medical	4.48	1.39	0.000	2.43	8.24
Intercept	0.00	0.01	0.000	0.00	0.02

**Table 3:** Multivariate logistic model for SRH of Veterans of Reserve Class A/B (*n* = 468)

Variables	Odds Ratio	Std. Err.	P	95% Confidence Interval	
				IL	UL
Education					
Less than HS	2.00	1.58	0.379	0.42	9.40
High School	4.30	2.55	0.012	1.37	13.81
Post sec < Bach	2.05	1.19	0.215	0.65	6.44
Arthritis					
Yes	6.60	3.76	0.001	2.15	20.23
Diabetes					
Yes	11.19	8.05	0.001	2.72	46.00
Obese					
Yes	3.37	1.54	0.008	1.37	8.27
Daily smoking					
Yes	2.98	1.56	0.039	1.05	8.38
Anxiety					
Yes	9.80	4.81	0.000	3.70	25.75
Intercept	0.01	0.01	0.000	0.00	0.03

The Reserve C model was assessed with 891 complete observations, and included 9 variables, of which three were common with the Reserve A/B model. Individuals who were single and had 50–59 years had higher odds for poor SRH than the reference category. Moreover, within the chronic conditions domain having self-reported symptoms of arthritis, back problems, and obesity presented higher odds of poor SRH than those who did not report any of these conditions. The mental health domain showed that depression, anxiety and PTSD were also associated with poor SRH. Within military service characteristics, enrollment and release type were also associated with poor SRH (Fig. 1, Supplementary Table 1), showing that Veterans released for medical reasons had the highest odds when compared with those who retired voluntarily.

The Reserve A/B model was assessed with 468 final observations, and included 6 variables in total, of which three were not used in the Reserve C model (Table 2). From the demographic dimension, having completed high school had higher odds of poor SRH. Moreover, from the physical and mental health dimensions having diabetes, obesity, being a daily smoker, and anxiety increased the odds for poor SRH.

## Discussion

Perceived health indicators, such as SRH, have been widely used in epidemiological research and it has been suggested that SRH reflects non-biologically detectable health conditions [20]. Moreover, SRH has been associated with lifestyle, psycho-social, and socio-demographic conditions [21], and a good predictor of future morbidity and mortality [22]. For the specific case of military personnel and veterans, determinants of SRH have been reported [10, 15]. Military occupation adds a level of complexity in the understanding of health, since armed forces operate in challenging environments with multiple “layers” of occupational hazards [14, 23]. Our results showed that SRH in Veterans of the Canadian Reserve Force can be affected at different magnitudes, by specific factors from the four domains of characteristics (demographics; physical health, mental health, and military service). Based on the knowledge of the authors, this is the first report of SRH amongst Veterans of the Canadian Reserve Force.

Veterans of both Reserve classes had different factors across the four domains related with the SRH outcome. Veterans of Reserve Class C who were single had a greater odds of poor SRH when compared with being married, but widowed or divorced were not statistically significant different from the reference category. This was consistent with findings of a population based study [23]. Poor SRH among these Veterans was significantly associated with older age, similar to other study among serving and ex-serving UK military personnel [11].

Veterans of Reserve Class A/B presented a reduced number of predictors, including demographic and health variables. Interestingly, age was not significant for these Veterans. This finding could be explained by their age distribution, since 56.5% were below 30 years. The relationship of lower education with poor SRH in these Veterans is consistent with international research that described an “education gradient” [24]. Since none of the military characteristics were associated with SRH, these Veterans appear to have similar SRH determinants with the general population. Release type may have been difficult to find associated, since most Veterans of Reserve Class A/B released for voluntary reasons, with very few medically discharged.

Reserve Force Veterans of the two classes had different chronic conditions associated with poor SRH. Veterans of Reserve Class C had SRH associated with several physical conditions (arthritis, back problems and obesity) and mental conditions (depression, anxiety and PTSD). On the other hand, Veterans of Reserve Class A/B had SRH associated with similar physical conditions of arthritis, diabetes and obesity and interestingly, only anxiety of the mental health conditions was significantly associated with SRH. The different conditions associated with SRH for both reserve classes could be a reflection of the nature of the class of Reserve service and its effects on SRH.

Chronic health conditions, mental health disorders and poor SRH are more frequent in Veterans of Reserve Class C than Canadians, and these Veterans have similar rates as Regular Force Veterans [18]. On the other hand, Veterans of Reserve Class A/B reported similar rates as the Canadian population [18]. One study in active Canadian regular force members, reported that chronic pain conditions were prevalent and co-occur with mental disorders [13]. The presence of one or more chronic health conditions has been associated with poor SRH in adult populations [25, 26], and specifically diabetes [26]. In our study, each separate chronic condition had an effect on poor SRH, and diabetes had the highest OR for Veterans of Reserve Class A/B, although the confidence interval of the OR was wide. The Reserve Class C had more co-occurring conditions with a higher prevalence of poor SRH, when compared with the Reserve Class A/B. These differences could help for health promotion in both Reserve Classes, stressing the importance of health programs and services during their transition to civilian life.

Amongst the military service characteristics of Veterans of Reserve Class C, being released from the service for medical reasons showed the highest odds of poor SRH, when compared to voluntary release (Voluntary/Retirement/Service Completed). This result was in agreement with an study amongst British Armed Forces, that showed a higher frequency of poor or fair SRH on medically downgraded individuals compared to those not medically downgraded [27], which were more likely to have psychological distress [27]. In contrast, type or release was not associated with SRH among Veterans of Reserve Class A/B.

One of the main strengths of this study is the large sample size, which was representative of Veterans of several Reserve Force classes. Moreover, this study used a large scale survey with objective measures of demographic, military and well-being characteristics using valid instruments. However, the study also has several limitations including the self-reported measures used for health, and the cross sectional nature of the survey, from which causal inference is limited.

## Conclusions

This study showed that specific domains of demography, chronic health conditions and military service can influence with different degrees of association the SRH. Poor SRH amongst Reserve Class C Veterans was associated with being single, being older and having chronic health conditions such as arthritis, back problems and obesity, or mental health conditions and being medically released. In contrast, fewer associations were found for Veterans with Reserve Class A/B service, showing a different risk composition that included completing at least high school, having a chronic health condition such as arthritis, diabetes and obesity, or anxiety were associated with poor SRH. In general the differences between the Reserve Classes provide insights in to demographic, health and well-being, that will help to inform policies and service delivery, depending on their specific needs derived from the nature of their service. This information can help to understand the relevant factors by Reserve class, that can contribute to improve the general health of Reserve Force Veterans.

## Supplementary information

**Additional file 1: Supplementary Material Table 1.** Bivariate analysis for SRH of Veterans of Reserve Class C, by demographic, physical health, mental health and military service characteristics, LASS 2013. Supplementary Material Table 2 Bivariate analysis for SRH of Veterans of Reserve Class A/B, by demographic, physical health, mental health and military service characteristics, LASS 2013.

## Data Availability

The database used in this report is available in the Research Data Centers operated by Statistics Canada. Further details can be found in the referenced technical report.

## Abbreviations

SRH: Self-rated health
LASS: Life after service
OR: Odds ratio
PTSD: Post traumatic stress disorder
NCM: Non-Commissioned Members
MOC: Military occupation
GOS: Generalized Operational Services
