# Supplementary material for: Characterization of occupational, demographic and health determinants in Canadian reservists veterans and the relationship with poor self-rated health
Source: Health Qual Life Outcomes. 2020 Aug 10;18:274. doi: 10.1186/s12955-020-01516-8 (PMC7418300; doi:10.1186/s12955-020-01516-8)
Supplement: Supplementary file 1 — Additional file 1: Supplementary Material Table 1. Bivariate analysis for SRH of Veterans of Reserve Class C, by demographic, physical health, mental health and military service characteristics, LASS 2013. Supplementary Material Table 2 Bivariate analysis for SRH of Veterans of Reserve Class A/B, by demographic, physical health, mental health and military service characteristics, LASS 2013. [file 12955_2020_1516_MOESM1_ESM.docx]

Supplementary Material Table 1. Bivariate analysis for SRH of Veterans of Reserve Class C, by demographic, physical health, mental health and military service characteristics, LASS 2013

| Domain | Variable | Categories | Class of Enrollment in Canadian Forces | | | | | | | |
| --- | --- | --- | --- | --- | --- | --- | --- | --- | --- | --- |
|  |  |  | Reserve Class C | | | | | | | P |
|  |  |  | SRH | | | | | | |  |
|  |  |  | Good/VG/EX | | | Fair/Poor | | Total | |  |
|  |  |  | n | % | n | | % | n | % |  |
| Demographics | Gender of respondent | Male | 614 | 77.23 | 89 | | 70.08 | 703 | 76.25 | ref |
|  |  | Female | 181 | 22.77 | 38 | | 29.92 | 219 | 23.75 | 0.075 |
|  |  | Total | 795 | 100.00 | 127 | | 100.00 | 922 | 100.00 |  |
|  | Age | <30 | 129 | 16.23 | 6 | | 4.72 | 135 | 14.64 | ref |
|  |  | 30-39 | 335 | 42.14 | 31 | | 24.41 | 366 | 39.70 | 0.132 |
|  |  | 40-49 | 148 | 18.62 | 36 | | 28.35 | 184 | 19.96 | 0.00 |
|  |  | 50-59 | 107 | 13.46 | 43 | | 33.86 | 150 | 16.27 | 0.00 |
|  |  | 60+ | 76 | 9.56 | 11 | | 8.66 | 87 | 9.44 | 0.028 |
|  |  | Total | 795 | 100.00 | 127 | | 100.00 | 922 | 100.00 |  |
|  | Education | Less than HS | 17 | 2.14 | 11 | | 8.73 | 28 | 3.04 | 0.00 |
|  |  | High School Grad | 193 | 24.28 | 41 | | 32.54 | 234 | 25.41 | 0.002 |
|  |  | Post Sec < Bach | 324 | 40.75 | 52 | | 41.27 | 376 | 40.83 | 0.026 |
|  |  | Post Sec >= Bach | 261 | 32.83 | 22 | | 17.46 | 283 | 30.73 | ref |
|  |  | Total | 795 | 100.00 | 126 | | 100.00 | 921 | 100.00 |  |
|  | Marital Status | Married/Common-Law | 598 | 75.22 | 75 | | 59.06 | 673 | 72.99 | ref |
|  |  | Sep/Wid/Div | 47 | 5.91 | 13 | | 10.24 | 60 | 6.51 | 0.016 |
|  |  | Single,Never Married | 150 | 18.87 | 39 | | 30.71 | 189 | 20.50 | 0.002 |
|  |  | Total | 795 | 100.00 | 127 | | 100.00 | 922 | 100.00 |  |
| Chronic Health Characteristics | Arthritis | Yes | 102 | 12.94 | 56 | | 44.09 | 158 | 17.27 | 0.00 |
|  |  | No | 686 | 87.06 | 71 | | 55.91 | 757 | 82.73 | ref |
|  |  | Total | 788 | 100.00 | 127 | | 100.00 | 915 | 100.00 |  |
|  | Back Problems | Yes | 216 | 27.24 | 81 | | 63.78 | 297 | 32.28 | 0.00 |
|  |  | No | 577 | 72.76 | 46 | | 36.22 | 623 | 67.72 | ref |
|  |  | Total | 793 | 100.00 | 127 | | 100.00 | 920 | 100.00 |  |
|  | Respiratory | Yes | 49 | 6.17 | 19 | | 15.20 | 68 | 7.40 | 0.001 |
|  |  | No | 745 | 93.83 | 106 | | 84.80 | 851 | 92.60 | ref |
|  |  | Total | 794 | 100.00 | 125 | | 100.00 | 919 | 100.00 |  |
|  | Diabetes | Yes | 30 | 3.78 | 20 | | 15.75 | 50 | 5.43 | 0.00 |
|  |  | No | 763 | 96.22 | 107 | | 84.25 | 870 | 94.57 | ref |
|  |  | Total | 793 | 100.00 | 127 | | 100.00 | 920 | 100.00 |  |
|  | Obese | Yes | 164 | 20.92 | 55 | | 44.35 | 219 | 24.12 | 0.00 |
|  |  | No | 620 | 79.08 | 69 | | 55.65 | 689 | 75.88 | ref |
|  |  | Total | 784 | 100.00 | 124 | | 100.00 | 908 | 100.00 |  |
|  | Cancer | Yes | 5 | 0.63 | 5 | | 3.94 | 10 | 1.09 | 0.003 |
|  |  | No | 788 | 99.37 | 122 | | 96.06 | 910 | 98.91 | ref |
|  |  | Total | 793 | 100.00 | 127 | | 100.00 | 920 | 100.00 |  |
|  | High Blood Pressure | Yes | 86 | 10.87 | 35 | | 27.78 | 121 | 13.20 | 0.00 |
|  |  | No | 705 | 89.13 | 91 | | 72.22 | 796 | 86.80 | ref |
|  |  | Total | 791 | 100.00 | 126 | | 100.00 | 917 | 100.00 |  |
|  | Hearing | Yes | 31 | 3.99 | 14 | | 12.17 | 45 | 5.04 | 0.00 |
|  |  | No | 746 | 96.01 | 101 | | 87.83 | 847 | 94.96 | ref |
|  |  | Total | 777 | 100.00 | 115 | | 100.00 | 892 | 100.00 |  |
|  | Smoker Daily | Yes | 92 | 11.57 | 28 | | 22.05 | 120 | 13.02 | 0.004 |
|  |  | No | 703 | 88.43 | 99 | | 77.95 | 802 | 86.98 | ref |
|  |  | Total | 795 | 100.00 | 127 | | 100.00 | 922 | 100.00 |  |
|  | Heavy Drinker | Yes | 215 | 27.25 | 29 | | 23.02 | 244 | 26.67 | 0.424 |
|  |  | No | 574 | 72.75 | 97 | | 76.98 | 671 | 73.33 | ref |
|  |  | Total | 789 | 100.00 | 126 | | 100.00 | 915 | 100.00 |  |
| Mental Health | Depression | Yes | 54 | 6.80 | 60 | | 47.24 | 114 | 12.38 | 0.00 |
|  |  | No | 740 | 93.20 | 67 | | 52.76 | 807 | 87.62 | ref |
|  |  | Total | 794 | 100.00 | 127 | | 100.00 | 921 | 100.00 |  |
|  | Anxiety | Yes | 35 | 4.41 | 41 | | 32.80 | 76 | 8.27 | 0.00 |
|  |  | No | 759 | 95.59 | 84 | | 67.20 | 843 | 91.73 | ref |
|  |  | Total | 794 | 100.00 | 125 | | 100.00 | 919 | 100.00 |  |
|  | PTSD | Yes | 31 | 3.93 | 40 | | 32.26 | 71 | 7.78 | 0.00 |
|  |  | No | 758 | 96.07 | 84 | | 67.74 | 842 | 92.22 | ref |
|  |  | Total | 789 | 100.00 | 124 | | 100.00 | 913 | 100.00 |  |
|  | Self-Perceived Mental Health | Excellent/VG | 587 | 73.93 | 31 | | 24.41 | 618 | 67.10 |  |
|  |  | Good | 174 | 21.91 | 26 | | 20.47 | 200 | 21.72 |  |
|  |  | Fair/Poor | 33 | 4.16 | 70 | | 55.12 | 103 | 11.18 |  |
|  |  | Total | 794 | 100.00 | 127 | | 100.00 | 921 | 100.00 |  |
| Military Characteristics | Length of service | < 2 years | 6 | 0.75 | 1 | | 0.79 | 7 | 0.76 | ref |
|  |  | 2 to 9 years | 308 | 38.74 | 30 | | 23.62 | 338 | 36.66 | 0.709 |
|  |  | 10 to 19 years | 303 | 38.11 | 49 | | 38.58 | 352 | 38.18 | 0.947 |
|  |  | >= 20 years | 178 | 22.39 | 47 | | 37.01 | 225 | 24.40 | 0.6 |
|  |  | Total | 795 | 100.00 | 127 | | 100.00 | 922 | 100.00 |  |
|  | Enrollment Era | 1950-80s | 194 | 24.40 | 55 | | 43.31 | 249 | 27.01 | ref |
|  |  | 1990s | 389 | 48.93 | 55 | | 43.31 | 444 | 48.16 | 0.001 |
|  |  | 2000-10s | 212 | 26.67 | 17 | | 13.39 | 229 | 24.84 | 0.00 |
|  |  | Total | 795 | 100.00 | 127 | | 100.00 | 922 | 100.00 |  |
|  | Rank | Officers | 148 | 18.62 | 21 | | 16.54 | 169 | 18.33 | ref |
|  |  | Senior NCM | 166 | 20.88 | 38 | | 29.92 | 204 | 22.13 | 0.093 |
|  |  | Junior NCM | 481 | 60.50 | 68 | | 53.54 | 549 | 59.54 | 0.909 |
|  |  | Total | 795 | 100.00 | 127 | | 100.00 | 922 | 100.00 |  |
|  | Environment | Air | 52 | 6.54 | 15 | | 11.81 | 67 | 7.27 | 0.051 |
|  |  | Land | 639 | 80.38 | 99 | | 77.95 | 738 | 80.04 | 0.595 |
|  |  | Sea | 104 | 13.08 | 13 | | 10.24 | 117 | 12.69 | ref |
|  |  | Total | 795 | 100.00 | 127 | | 100.00 | 922 | 100.00 |  |
|  | Release Type | Involuntary | 62 | 7.80 | 7 | | 5.51 | 69 | 7.48 | 0.437 |
|  |  | Medical | 64 | 8.05 | 64 | | 50.39 | 128 | 13.88 | 0.00 |
|  |  | Vol/Ret/Service Complete | 669 | 84.15 | 56 | | 44.09 | 725 | 78.63 | ref |
|  |  | Total | 795 | 100.00 | 127 | | 100.00 | 922 | 100.00 |  |
|  | Military occupation | Combat arms | 246 | 44.40 | 36 | | 32.73 | 282 | 42.47 | 0.004 |
|  |  | Communications | 62 | 11.19 | 9 | | 8.18 | 71 | 10.69 | 0.062 |
|  |  | Maritime | 45 | 8.12 | 6 | | 5.45 | 51 | 7.68 | 0.113 |
|  |  | Aviation | 10 | 1.81 | 1 | | 0.91 | 11 | 1.66 | 0.293 |
|  |  | Admin/logistics | 144 | 25.99 | 43 | | 39.09 | 187 | 28.16 | ref |
|  |  | Engineering/technical | 21 | 3.79 | 6 | | 5.45 | 27 | 4.07 | 0.895 |
|  |  | Medical | 19 | 3.43 | 8 | | 7.27 | 27 | 4.07 | 0.304 |
|  |  | GOS | 7 | 1.26 | 1 | | 0.91 | 8 | 1.20 | 0.526 |
|  |  | Total | 554 | 100.00 | 110 | | 100.00 | 664 | 100.00 |  |

Supplementary Material Table 2. Bivariate analysis for SRH of Veterans of Reserve Class A/B, by demographic, physical health, mental health and military service characteristics, LASS 2013.

| Domain | Variable | Categories | Class of Enrollment in Canadian Forces | | | | | | |
| --- | --- | --- | --- | --- | --- | --- | --- | --- | --- |
|  |  |  | Reserve Class A/B | | | | | | P |
|  |  |  | SRH | | | | | |  |
|  |  |  | Good/VG/EX | | Fair/Poor | | Total | |  |
|  |  |  | n | % | n | % | n | % |  |
| Demographics | Gender of respondent | Male | 363 | 82.31 | 24 | 68.57 | 387 | 81.30 | ref |
|  |  | Female | 78 | 17.69 | 11 | 31.43 | 89 | 18.70 | 0.059 |
|  |  | Total | 441 | 100.00 | 35 | 100.00 | 476 | 100.00 |  |
|  | Age | <30 | 238 | 53.97 | 15 | 42.86 | 253 | 53.15 | ref |
|  |  | 30-39 | 132 | 29.93 | 7 | 20.00 | 139 | 29.20 | 0.469 |
|  |  | 40-49 | 45 | 10.20 | 5 | 14.29 | 50 | 10.50 | 0.334 |
|  |  | 50-59 | 12 | 2.72 | 5 | 14.29 | 17 | 3.57 | 0.003 |
|  |  | 60+ | 14 | 3.17 | 3 | 8.57 | 17 | 3.57 | 0.071 |
|  |  | Total | 441 | 100.00 | 35 | 100.00 | 476 | 100.00 |  |
|  | Education | Less than HS | 7 | 1.59 | 3 | 8.57 | 10 | 2.11 | 0.002 |
|  |  | High School Grad | 101 | 22.95 | 14 | 40.00 | 115 | 24.21 | 0.008 |
|  |  | Post Sec < Bach | 169 | 38.41 | 13 | 37.14 | 182 | 38.32 | 0.097 |
|  |  | Post Sec >= Bach | 163 | 37.05 | 5 | 14.29 | 168 | 35.37 | ref |
|  |  | Total | 440 | 100.00 | 35 | 100.00 | 475 | 100.00 |  |
|  | Marital Status | Married/Common-Law | 248 | 56.36 | 24 | 68.57 | 272 | 57.26 | ref |
|  |  | Sep/Wid/Div | 19 | 4.32 | 2 | 5.71 | 21 | 4.42 | 0.956 |
|  |  | Single,Never Married | 173 | 39.32 | 9 | 25.71 | 182 | 38.32 | 0.178 |
|  |  | Total | 440 | 100.00 | 35 | 100.00 | 475 | 100.00 |  |
| Chronic Health Characteristics | Arthritis | Yes | 19 | 4.32 | 12 | 34.29 | 31 | 6.53 | 0.00 |
|  |  | No | 421 | 95.68 | 23 | 65.71 | 444 | 93.47 | ref |
|  |  | Total | 440 | 100.00 | 35 | 100.00 | 475 | 100.00 |  |
|  | Back Problems | Yes | 67 | 15.19 | 17 | 48.57 | 84 | 17.65 | 0.00 |
|  |  | No | 374 | 84.81 | 18 | 51.43 | 392 | 82.35 | ref |
|  |  | Total | 441 | 100.00 | 35 | 100.00 | 476 | 100.00 |  |
|  | Respiratory | Yes | 24 | 5.45 | 2 | 5.71 | 26 | 5.47 | 0.949 |
|  |  | No | 416 | 94.55 | 33 | 94.29 | 449 | 94.53 | ref |
|  |  | Total | 440 | 100.00 | 35 | 100.00 | 475 | 100.00 |  |
|  | Diabetes | Yes | 3 | 0.68 | 5 | 14.29 | 8 | 1.68 | 0.00 |
|  |  | No | 438 | 99.32 | 30 | 85.71 | 468 | 98.32 | ref |
|  |  | Total | 441 | 100.00 | 35 | 100.00 | 476 | 100.00 |  |
|  | Obese | Yes | 71 | 16.25 | 15 | 44.12 | 86 | 18.26 | 0 |
|  |  | No | 366 | 83.75 | 19 | 55.88 | 385 | 81.74 | ref |
|  |  | Total | 437 | 100.00 | 34 | 100.00 | 471 | 100.00 |  |
|  | Cancer | Yes | 3 | 0.68 | 1 | 2.86 | 4 | 0.84 | 0.145 |
|  |  | No | 437 | 99.32 | 34 | 97.14 | 471 | 99.16 | ref |
|  |  | Total | 440 | 100.00 | 35 | 100.00 | 475 | 100.00 |  |
|  | High Blood Pressure | Yes | 21 | 4.79 | 12 | 34.29 | 33 | 6.98 | 0.00 |
|  |  | No | 417 | 95.21 | 23 | 65.71 | 440 | 93.02 | ref |
|  |  | Total | 438 | 100.00 | 35 | 100.00 | 473 | 100.00 |  |
|  | Hearing | Yes | 5 | 1.14 | 2 | 5.88 | 7 | 1.49 | 0.098 |
|  |  | No | 432 | 98.86 | 32 | 94.12 | 464 | 98.51 | ref |
|  |  | Total | 437 | 100.00 | 34 | 100.00 | 471 | 100.00 |  |
|  | Smoker Daily | YEs | 41 | 9.30 | 8 | 22.86 | 49 | 10.29 | 0.013 |
|  |  | No | 400 | 90.70 | 27 | 77.14 | 427 | 89.71 | ref |
|  |  | Total | 441 | 100.00 | 35 | 100.00 | 476 | 100.00 |  |
|  | Heavy Drinker | Yes | 141 | 32.19 | 8 | 23.53 | 149 | 31.57 | 0.345 |
|  |  | No | 297 | 67.81 | 26 | 76.47 | 323 | 68.43 | ref |
|  |  | Total | 438 | 100.00 | 34 | 100.00 | 472 | 100.00 |  |
| Mental Health | Depression | Yes | 17 | 3.86 | 12 | 34.29 | 29 | 6.11 | 0.00 |
|  |  | No | 423 | 96.14 | 23 | 65.71 | 446 | 93.89 | ref |
|  |  | Total | 440 | 100.00 | 35 | 100.00 | 475 | 100.00 |  |
|  | Anxiety | Yes | 18 | 4.09 | 8 | 22.86 | 26 | 5.47 | 0.00 |
|  |  | No | 422 | 95.91 | 27 | 77.14 | 449 | 94.53 | ref |
|  |  | Total | 440 | 100.00 | 35 | 100.00 | 475 | 100.00 |  |
|  | PTSD | Yes | 4 | 0.91 | 5 | 15.15 | 9 | 1.90 | 0.00 |
|  |  | No | 436 | 99.09 | 28 | 84.85 | 464 | 98.10 | ref |
|  |  | Total | 440 | 100.00 | 33 | 100.00 | 473 | 100.00 |  |
|  | Self-Perceived Mental Health | Excellent/VG | 337 | 76.42 | 11 | 31.43 | 348 | 73.11 |  |
|  |  | Good | 87 | 19.73 | 10 | 28.57 | 97 | 20.38 |  |
|  |  | Fair/Poor | 17 | 3.85 | 14 | 40.00 | 31 | 6.51 |  |
|  |  | Total | 441 | 100.00 | 35 | 100.00 | 476 | 100.00 |  |
| Military Characteristics | Length of Service | < 2 years | 91 | 20.63 | 4 | 11.43 | 95 | 19.96 | ref |
|  |  | 2 to 9 years | 286 | 64.85 | 20 | 57.14 | 306 | 64.29 | 0.398 |
|  |  | 10 to 19 years | 45 | 10.20 | 10 | 28.57 | 55 | 11.55 | 0.015 |
|  |  | >= 20 years | 19 | 4.31 | 1 | 2.86 | 20 | 4.20 | 0.951 |
|  |  | Total | 441 | 100.00 | 35 | 100.00 | 476 | 100.00 |  |
|  | Enrollment era | 1950-80s | 23 | 5.22 | 2 | 5.71 | 25 | 5.25 | ref |
|  |  | 1990s | 74 | 16.78 | 12 | 34.29 | 86 | 18.07 | 0.501 |
|  |  | 2000-10s | 344 | 78.00 | 21 | 60.00 | 365 | 76.68 | 0.703 |
|  |  | Total | 441 | 100.00 | 35 | 100.00 | 476 | 100.00 |  |
|  | Rank | Officers | 49 | 11.11 | 2 | 5.71 | 51 | 10.71 | ref |
|  |  | Senior NCM | 16 | 3.63 | 6 | 17.14 | 22 | 4.62 | 0.012 |
|  |  | Junior NCM | 376 | 85.26 | 27 | 77.14 | 403 | 84.66 | 0.485 |
|  |  | Total | 441 | 100.00 | 35 | 100.00 | 476 | 100.00 |  |
|  | Environment | Air | 12 | 2.72 | 7 | 20.00 | 19 | 3.99 | 0.004 |
|  |  | Land | 369 | 83.67 | 25 | 71.43 | 394 | 82.77 | 0.729 |
|  |  | Sea | 60 | 13.61 | 3 | 8.57 | 63 | 13.24 | ref |
|  |  | Total | 441 | 100.00 | 35 | 100.00 | 476 | 100.00 |  |
|  | Release Type | Involuntary | 57 | 12.98 | 5 | 14.29 | 62 | 13.08 | 0.414 |
|  |  | Medical | 9 | 2.05 | 8 | 22.86 | 17 | 3.59 | 0.00 |
|  |  | Vol/Ret/Service Complete | 373 | 84.97 | 22 | 62.86 | 395 | 83.33 | ref |
|  |  | Total | 439 | 100.00 | 35 | 100.00 | 474 | 100.00 |  |
|  | Military occupation | Combat arms | 211 | 58.45 | 15 | 48.39 | 226 | 57.65 | 0.346 |
|  |  | Communications | 26 | 7.20 | 0 | 0.00 | 26 | 6.63 | -- |
|  |  | Maritime | 36 | 9.97 | 3 | 9.68 | 39 | 9.95 | 0.743 |
|  |  | Aviation | 3 | 0.83 | 1 | 3.23 | 4 | 1.02 | 0.34 |
|  |  | Admin/logistics/ | 53 | 14.68 | 7 | 22.58 | 60 | 15.31 | ref |
|  |  | Engineering/technical | 7 | 1.94 | 2 | 6.45 | 9 | 2.30 | 0.267 |
|  |  | Medical | 20 | 5.54 | 3 | 9.68 | 23 | 5.87 | 0.846 |
|  |  | GOS | 5 | 1.39 | 0 | 0.00 | 5 | 1.28 | -- |
|  |  | Total | 361 | 100.00 | 31 | 100.00 | 392 | 100.00 |  |
